# The vulnerability of calretinin-containing hippocampal interneurons to temporal lobe epilepsy

**DOI:** 10.3389/fnana.2014.00100

**Published:** 2014-09-29

**Authors:** Kinga Tóth, Zsófia Maglóczky

**Affiliations:** ^1^Institute of Cognitive Neuroscience and Psychology, Research Centre for Natural Sciences, Hungarian Academy of SciencesBudapest, Hungary; ^2^Institute of Experimental Medicine, Hungarian Academy of SciencesBudapest, Hungary

**Keywords:** calretinin, interneuron, dendritic inhibition, synchronization, epilepsy

## Abstract

This review focuses on the vulnerability of a special interneuron type—the calretinin (CR)-containing interneurons—in temporal lobe epilepsy (TLE). CR is a calcium-binding protein expressed mainly by GABAergic interneurons in the hippocampus. Despite their morphological heterogeneity, CR-containing interneurons form a distinct subpopulation of inhibitory cells, innervating other interneurons in rodents and to some extent principal cells in the human. Their dendrites are strongly connected by zona adherentiae and presumably by gap junctions both in rats and humans. CR-containing interneurons are suggested to play a key role in the hippocampal inhibitory network, since they can effectively synchronize dendritic inhibitory interneurons. The sensitivity of CR-expressing interneurons to epilepsy was discussed in several reports, both in animal models and in humans. In the sclerotic hippocampus the density of CR-immunopositive cells is decreased significantly. In the non-sclerotic hippocampus, the CR-containing interneurons are preserved, but their dendritic tree is varicose, segmented, and zona-adherentia-type contacts can be less frequently observed among dendrites. Therefore, the dendritic inhibition of pyramidal cells may be less effective in TLE. This can be partially explained by the impairment of the CR-containing interneuron ensemble in the epileptic hippocampus, which may result in an asynchronous and thus less effective dendritic inhibition of the principal cells. This phenomenon, together with the sprouting of excitatory pathway axons and enhanced innervation of principal cells, may be involved in seizure generation. Preventing the loss of CR-positive cells and preserving the integrity of CR-positive dendrite gap junctions may have antiepileptic effects, maintaining proper inhibitory function and helping to protect principal cells in epilepsy.

## Introduction

Calretinin (CR) is a calcium-bindig protein, belonging to the calmodulin superfamily, which was shown to be present in many brain regions (Rogers, 1987; Faas et al., 2007).

In the rodent hippocampus the majority of the CR-positive cells seem to be GABAergic interneurons (Jacobowitz and Winsky, 1991; Miettinen et al., 1992; Liu et al., 1996).

They represent a distinct subpopulation of interneurons (Gulyás et al., 1992; Liu et al., 1996) with a negligible overlap with other calcium binding protein-containing interneurons—parvalbumin and calbindin—in rat and monkey (Miettinen et al., 1992; Rogers and Resibois, 1992; Seress et al., 1993b).

Interneurons of the hippocampus can be divided into three main functional groups according to their role in the neuronal network (Freund and Buzsáki, 1996). Perisomatic inhibitory cells innervate the soma, axon initial segment or proximal dendrites of principal cells (basket and axo-axonic cells) (Handelmann et al., 1981; Emson et al., 1982; Somogyi et al., 1983; Kosaka et al., 1985, 1987; Katsumaru et al., 1988; Seress et al., 1991, 1993a; Li et al., 1992; Ribak et al., 1993; Halasy et al., 1996) and control the output of principal cells (Arai et al., 1995; Freund and Buzsáki, 1996; Miles et al., 1996; Holmes and Levy, 1997). Dendritic inhibitory cells innervate the distal dendrites of principal cells (Kawaguchi and Hama, 1988; Gulyás et al., 1993; Han et al., 1993; Buhl et al., 1994; Sik et al., 1994, 1995, 1997; Buckmaster and Schwartzkroin, 1995; Halasy et al., 1996) and control the generation of dendritic calcium spikes and synaptic plasticity (Freund and Buzsáki, 1996; Miles et al., 1996). The interneuron-selective inhibitory cells innervate other interneurons, and thus have a role in the synchronization of dendritic inhibition (Acsády et al., 1996; Gulyás et al., 1996; Hajos et al., 1996; Urbán et al., 2002).

The different vulnerability of interneurons in temporal lobe epilepsy (TLE) was shown in numerous animal models and human patients (Babb et al., 1989; Houser, 1991; Sloviter, 1999; Ben-Ari and Cossart, 2000; Bouilleret et al., 2000; André et al., 2001; Ben-Ari, 2001; Arellano et al., 2004; Ben-Ari and Holmes, 2005; Kuruba et al., 2011; Marx et al., 2013). In most studies, the relative preservation of the inhibitory input to the perisomatic domain of principal cells was described, whereas dendritic inhibition was found to be decreased (Cossart et al., 2001; Sundstrom et al., 2001; Wittner et al., 2001, 2005; Maglóczky and Freund, 2005; Tyan et al., 2014). The third functional type of interneurons are specialized to innervate other interneurons (Freund and Buzsáki, 1996; Gulyás et al., 1996) and contain VIP or CR (Acsády et al., 1996; Gulyás et al., 1996). These neurons are in an ideal position to regulate dendritic inhibition (Gulyás et al., 1996; Hajos et al., 1996; Chamberland et al., 2010; Tyan et al., 2014) and compensate the synchronizing effect of perisomatic inhibition (Cossart et al., 2001; Maglóczky and Freund, 2005), and therefore they may have a critic role in maintaining the normal network activity and may prevent synchronous discharges leading to epileptic seizures.

In this review we focus on the fate of this special interneuron type—the CR-containing interneurons—in TLE.

## CR-containing cells in the rodent hippocampus

Two types of CR-positive cells were found in the rat hippocampus (Gulyás et al., 1992), spiny and spine-free dendritic cells. The spine-free type can be observed in all subregions, and have a small cell body with smooth dendrites running through several layers. Their dendrites are often attached to each other over long segments. At the electron microscopic level, several puncta adherentiae were observed among contacting CR-positive dendrites (Gulyás et al., 1992, 1996). The other, spiny type is found exclusively in the hilus of the dentate gyrus and in the stratum lucidum of the CA3 region. Their dendrites run horizontally and are covered with spines. They receive the majority of their inputs from mossy fibers (Gulyás et al., 1992). Miettinen and colleagues have shown that the majority of the former type belonged to GABAergic interneurons, whereas the latter, spiny type was mainly GABA-negative (Miettinen et al., 1992).

Hippocampal CR-positive cells of the mouse are similar to the rat. However, there are species-specific differences among human/rat and mouse hippocampal areas, i.e., young granule cells and mossy cells are CR-positive in mice, whereas they are CR-negative in rats and humans (Liu et al., 1996; Blasco-Ibáñez and Freund, 1997; Fujise et al., 1998; Murakawa and Kosaka, 1999; Mátyás et al., 2004; Seress et al., 2008). However, the main interneuron types, e.g., the bipolar or bitufted, rarely spiny cells with dendro-dendritic connections, are present both in rodents and humans in hippocampal areas (Gulyás et al., 1992; Nitsch and Ohm, 1995; Urbán et al., 2002). This cell type is thought to be responsible for the inhibition of other interneurons (Gulyás et al., 1996). It is well preserved throughout evolution, and therefore its role can be studied in rodent models of epilepsy.

## CR-containing cells in the human hippocampus

In the adult human hippocampus, CR is expressed by non-principal cells (Nitsch and Ohm, 1995; Urbán et al., 2002). Besides the interneurons, there are a few remaining Cajal-Retzius cells at the border of stratum moleculare and stratum lacunosum-moleculare that also show CR-immunoreactivity (Abraham and Meyer, 2003). Unlike in mice and to some extent in non-human primates, mossy cells of the human dentate gyrus are negative for CR-immunostaining (Maglóczky et al., 2000; Seress et al., 2008).

**Figure 1:**
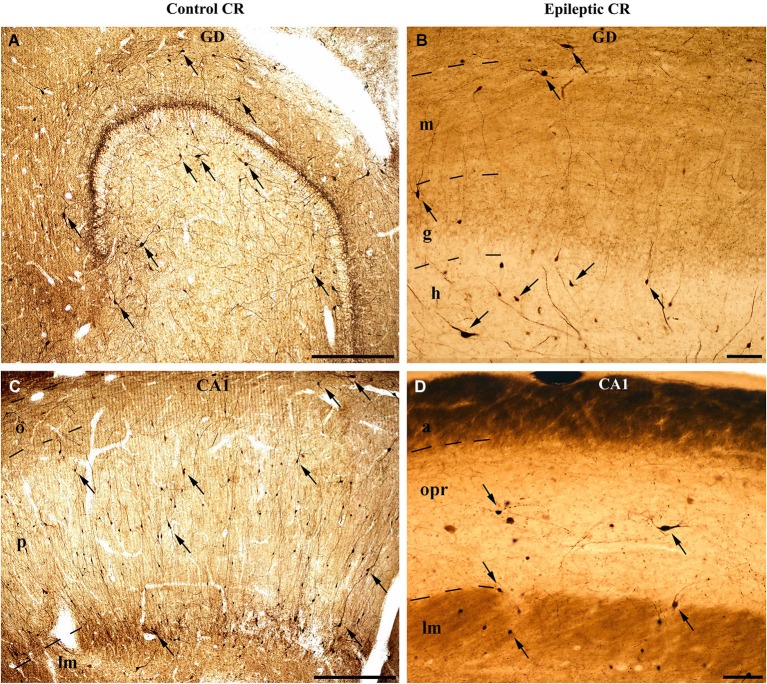
**Light micrographs show the distribution and density of CR-containing interneurons in the control (A, C) and epileptic human hippocampus (B, D)**. CR-immunopositive cells can be observed in the entire hippocampus. **(A)** They are present in large numbers in the control dentate gyrus, especially in the hilus. The immunopositive cells in the outer part of the molecular layer near the hippocampal fissure are presumably Cajal-Retzius cells. **(B)** The epileptic dentate gyrus contains less immunopositive cells. **(C)** Many CR-positive interneurons can be seen in the control CA1 region, scattered throughout all layers. The largest amount of cells is present in the stratum radiatum and at the border of stratum lacunosum-moleculare. **(D)** The sclerotic epileptic CA1 region contains only a few CR-immunopositive interneurons with short and distorted, often segmented dendrites. GD: dentate gyrus; m: stratum moleculare; g: stratum granulosum; h: hilus; CA1: Cornu Ammonis 1; o: stratum oriens; p: stratum pyramidale; lm: stratum lacunosum-moleculare; opr: strata oriens-pyramidale-radiatum. Scales: 500 μm in **(A)** and **(C)**; 100 μm in **(B)** and **(D)**.

**Figure 2:**
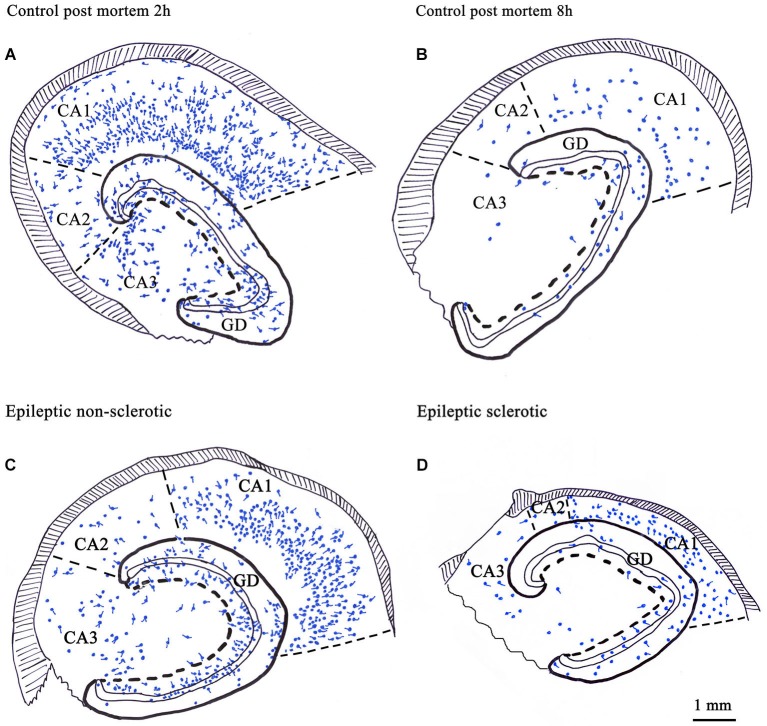
**Camera lucida drawings show the number and distribution of CR-immunoreactive cells in control human hippocampi with different post mortem delay (A, B) and in epileptic human hippocampi with different degrees of principal cell death (C, D)**. CR-containing cells were present in all regions of the hippocampus **(A)**. Their number was significantly reduced in control samples with long post mortem delay **(B)** and in the sclerotic epileptic cases **(D)**. In the non-sclerotic tissues **(C)** the number and distribution of CR-immunoreactive cells were similar to the control, with short post mortem delay **(A)**. The volume of the sclerotic hippocampi is reduced because of the shrinkage of the CA1 region due to the loss of CA1 pyramidal cells **(D)**. GD: dentate gyrus; Scale: 1 mm.

The distribution of the CR-positive elements shows the typical distribution of a non-perisomatic inhibitory interneuron type. They are abundant in the stratum radiatum and at the border of lacunosum-moleculare in the Cornu Ammonis and in the hilus of the dentate gyrus (Figures 1, 2; Nitsch and Ohm, 1995; Maglóczky et al., 2000; Urbán et al., 2002).

The human CR-containing interneurons form a morphologically heterogeneous cell population: (i) multipolar or fusiform cells in the hilus, with dendrites restricted mainly in this subregion; (ii) fusiform cells in the strata moleculare and oriens with horizontal dendrites; (iii) multipolar cells in all layers; and (iv) a group of small cells with a few short dendrites in the dentate gyrus (Nitsch and Ohm, 1995; Maglóczky et al., 2000; Urbán et al., 2002; Tóth et al., 2010).

The human CR-positive interneuron population differs somewhat from those in the rat and mouse (Murakawa and Kosaka, 1999; Mátyás et al., 2004). In human, there is an abundant group of multipolar CR-immunoreactive interneurons at the border of the CA1 stratum lacunosum-moleculare, and a group of small CR-positive cells in the dentate gyrus (Nitsch and Ohm, 1995), which are absent in the rat. In addition, the characteristic spiny CR-positive cells of the rat CA3 region (Gulyás et al., 1992) are missing in the human (Nitsch and Ohm, 1995; Urbán et al., 2002).

**Figure 3:**
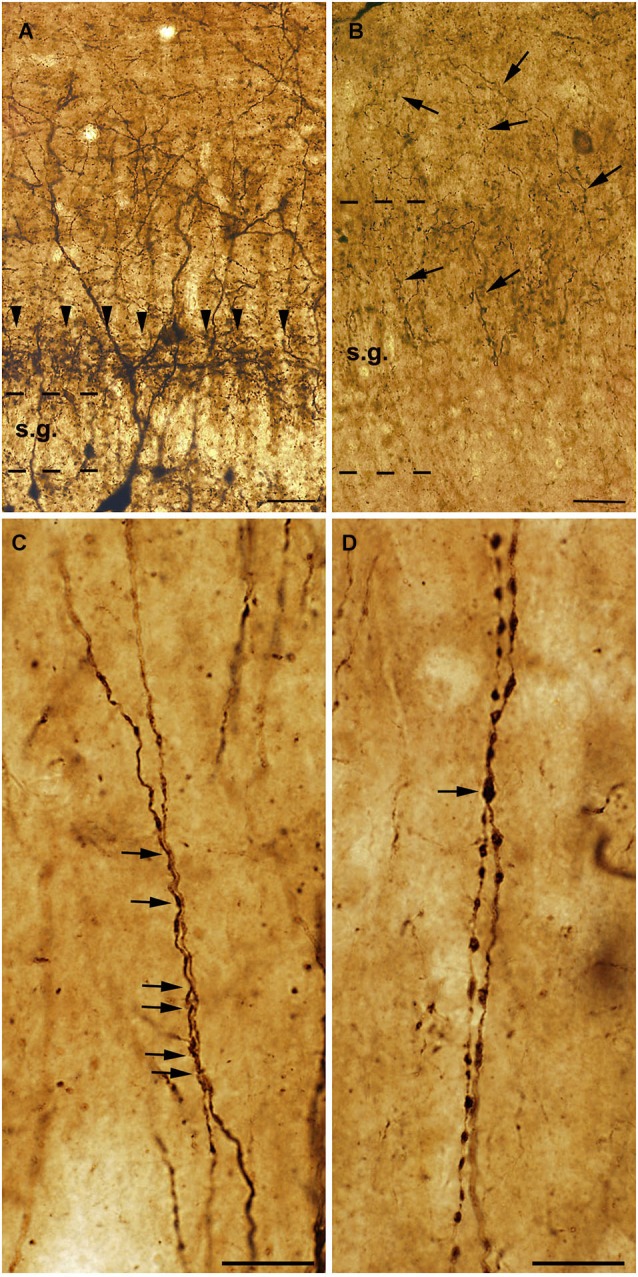
**High power light micrographs from control and epileptic samples showing the morphology of CR-positive interneuron dendrites**. Many CR-positive interneurons are located in the hilus of the control dentate gyrus **(A)**. They usually have long and smooth dendrites arborizing densely in the stratum moleculare. In the inner molecular layer a dense network of CR-positive axon terminals can be observed, which presumably originate from the supramammillary nucleus (**A**, arrowheads). In the sclerotic epileptic gyrus dentatus **(B)** most of the CR cells disappear, and the CR-positive supramammillary input extends to the entire stratum moleculare (**B**, arrows). The dispersion of the granule cell layer can also be observed in many epileptic cases. In the control CA1 region **(C)**, dendrites of CR-positive interneurons are usually long, smooth and vertically oriented. They are often juxtaposed and run together over short segments (**C**, arrows). However, in the non-sclerotic samples **(D)** the dendrites are varicose and the contacts between CR-positive interneuron dendrites are less frequent than in controls (**D**, arrow). s.g.: stratum granulosum; Scales: **(A, B)**: 50 μm; **(C, D)**: 20 μm.

The dendrites of the human CR-positive interneurons are smooth or rarely spiny. Similarly to rats, long segments of CR-positive dendrites of different cells are often attached to each other, especially in the CA1 region (Figure 3; Urbán et al., 2002). Zona- or puncta adherentia-type contacts were observed between these dendrites at the electron microscopic level (Urbán et al., 2002; Tóth et al., 2010).

## Extrinsic CR-containing network

Besides the intrinsic hippocampal GABAergic CR-system deriving from the local CR-containing inhibitory interneurons, there are extrinsic glutamatergic inputs that also contain CR. In rats, monkeys and humans, a dense CR-positive fiber network can be observed at the top of the granule cell layer in the inner third of the stratum moleculare (Gulyás et al., 1992; Nitsch and Léránth, 1993; Nitsch and Ohm, 1995). The vast majority of the CR-positive terminals here are putative excitatory terminals with a thick postsynaptic density (Gulyás et al., 1992; Maglóczky et al., 2000). This excitatory pathway was shown to be originating from the supramammillary nucleus both in rats and monkeys (Nitsch and Léránth, 1993; Maglóczky et al., 1994; Borhegyi and Leranth, 1997) and, besides the stratum moleculare, it also innervates the pyramidal layer of the CA2 region (Nitsch and Léránth, 1993; Maglóczky et al., 1994; Nitsch and Ohm, 1995). Finally, a dense CR-positive axonal network can be seen at the border of strata radiatum and lacunosum-moleculare in the CA1 region, with numerous presumably excitatory CR-positive axon terminals giving asymmetric synapses with thick postsynaptic densities (Urbán et al., 2002). In rats and monkeys, this pathway was shown to originate from the thalamic reunions nucleus (Amaral and Cowan, 1980; Fortin et al., 1996; Bokor et al., 2002; Drexel et al., 2011).

## Function of CR-containing interneurons in the hippocampus

The CR-positive interneurons are interneuron-selective inhibitory cells in the CA1 region of the rat hippocampus (Gulyás et al., 1996). Meskenaite have shown that the postsynaptic targets were partially GABAergic local circuit neurons in the monkey neocortex (Meskenaite, 1997). According to Urbán et al. a large proportion of these cells also belong to this functional type of interneuron in the human hippocampal CA1 region, innervating other CR-containing dendrites or unstained interneuron dendrites (Urbán et al., 2002). Additionally, in humans, there is a population which innervates the dendrites of the pyramidal cells (dendritic inhibitory interneurons) (Urbán et al., 2002).

**Figure 4:**
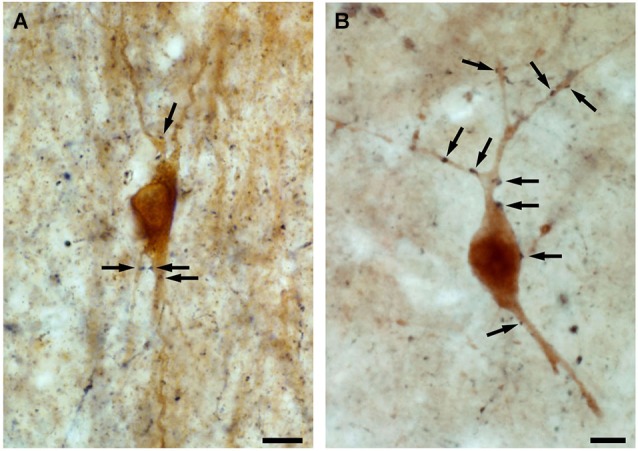
**High power light micrographs showing the innervation of calbindin-containing interneurons by CR-positive fibres.** By double immunolabeling experiments, applying DAB and DAB-Ni as chromogens, we show that both in the control (A) and in the epileptic (B) human CA1 region, the axon terminals of the CR-containing interneurons (black reaction product) innervate the calbindin-containing interneurons (brown reaction product) (arrows). Scale: 10 μm.

Interneuron-selective cells were suggested to be important in the synchronization of dendritic inhibitory cells (Gulyás et al., 1996; Maglóczky and Freund, 2005; Chamberland et al., 2010; Tyan et al., 2014). This is supported by the fact that: (i) their dendrites are strongly connected by zona adherentiae and possibly by gap junctions in rats (Gulyás et al., 1992, 1993), which allows the synchronous activation of the connected cell population (Galarreta and Hestrin, 1999; Gibson et al., 1999); (ii) their dendrites often run parallel with each other in the human CA1 region, forming close contacts; additionally, zona adherentiae were identified in these segments at the electron microscopic level (Urbán et al., 2002), which is a characteristic structure to support the development of gap junctions (Kosaka and Hama, 1985); and (iii) the calbindin-containing dendritic inhibitory interneurons are innervated by CR-positive inhibitory cells in both rats and humans (Figure 4; Gulyás et al., 1996; Tóth et al., 2010).

Dendritic inhibitory interneurons innervate the distal dendritic tree of pyramidal cells. The synchronization of dendritic inhibitory cells is a crucial process to provide an effective inhibitory control of excitatory synaptic input of pyramidal cell dendrites (Miles et al., 1996; Chamberland et al., 2010; Tyan et al., 2014).

These observations suggest that CR-containing cells form a unique inhibitory cell population in the hippocampus. Despite their low number, they play a key role in hippocampal inhibitory circuits. By synchronizing the dendritic inhibitory interneurons, they can control the efficacy of excitatory inputs to pyramidal cells (Maglóczky and Freund, 2005; Tyan et al., 2014). Therefore, studying their fate in different brain disorders, especially in epilepsy, is of special interest.

## Changes in the number and distribution of CR-positive interneurons in the human epileptic hippocampus

The sensitivity of CR-expressing cells to epilepsy was discussed in several reports, both in animal models and in humans (Maglóczky and Freund, 2005; Barinka and Druga, 2010; Tóth et al., 2010). CR-containing cells were also found to be sensitive in focal cortical dysplasias (Barinka et al., 2010).

However, the published data on the sensitivity of human CR-containing cells in epilepsy is controversial. According to Blümcke et al. the CR-containing cells are preserved in epilepsy (Blümcke et al., 1996); moreover, the number of CR-positive Cajal-Retzius cells is even increased (Blümcke et al., 1999; Thom et al., 2002). However, our group observed increased vulnerability of CR-positive cells in human TLE, including the CR-positive Cajal-Retzius cells (Figure 1; Maglóczky et al., 2000; Tóth et al., 2010).

The contradiction possibly emerged from the sensitivity of CR-immunostaining to the length of the post mortem delay of the applied control samples (Figure 2). The post mortem delay in the studies of Blümcke et al. varied between 6 h and up to 3 days (Blümcke et al., 1996, 1999). In our studies, post mortem delays for control samples were between 2–4 h (Maglóczky et al., 2000; Tóth et al., 2010). According to Urbán et al. the age of the subject, the post mortem delay and the fixation procedure has an extreme impact on the quality and quantity of CR-immunostaining (Urbán et al., 2002). The preservation of the immersion-fixed control samples with short post mortem delays was comparable to the perfusion-fixed animal tissue and immediately fixed epileptic samples (Tóth et al., 2010).

According to our results, the number of CR-containing cells decreased significantly, both in the dentate gyrus (especially in the hilus) (Maglóczky et al., 2000; Tóth et al., 2010) and in the sclerotic CA1 region (Figures 1, 2; Tóth et al., 2010). The density of the presumably persisting CR-positive Cajal-Retzius cells (Abraham and Meyer, 2003) at the border of the stratum moleculare and hippocampal fissure was also significantly reduced (Haas et al., 2002; Tóth et al., 2010), even in the non-sclerotic samples (Tóth et al., 2010).

In addition, the preserved cells had an altered morphology, suggesting the degeneration of their dendritic tree (Maglóczky et al., 2000; Tóth et al., 2010; Figure 3). In the non-sclerotic epileptic CA1 region, the number of CR-positive cells was unchanged. However, their dendrites were varicose, and contacts between the CR-positive dendrites were less frequently seen (Tóth et al., 2010; Figure 3). Consistently, zona adherentia-type contacts were rarely observed between CR-positive dendritic profiles at the electron microscopic level (Tóth et al., 2010), decreasing the possibility of the establishment and maintenance of gap junctions (Fukuda and Kosaka, 2000).

Thus, the synchronous activation of these interneuron-selective inhibitory cells is possibly impaired in the human epileptic hippocampus. As part of the CR-containing cells are dendritic inhibitory interneurons in humans, this means that by losing their zona adherentia-type dendro-dendritic contacts, the CR-positive population of dendritic inhibitory cells also loses the ability to function synchronously.

### Synaptic reorganization of CR-positive interneurons in the human epileptic DG and CA1

Electron microscopic examinations revealed that the CR-containing interneuronal (inhibitory) synaptic network is also changed in the epileptic human samples.

Despite the sensitivity and loss of CR-containing interneurons in the DG (Maglóczky et al., 2000; Tóth et al., 2010), an increased frequency of CR-positive interneuronal terminals was found in epilepsy (Maglóczky et al., 2000). Dentate granule cells receive an excess excitation in epilepsy due to mossy fiber sprouting (Davenport et al., 1990b; Houser et al., 1990; Represa et al., 1990; Sloviter, 1994; Mathern et al., 1995; Zhang et al., 2009). Although the increase of the frequency of CR-positive inhibitory terminals was low, regarding the severe cell loss, this might mean a sprouting of the remaining CR-containing inhibitory cells as a compensatory mechanism to offset the enhanced excitatory input on granule cell dendrites.

**Figure 5:**
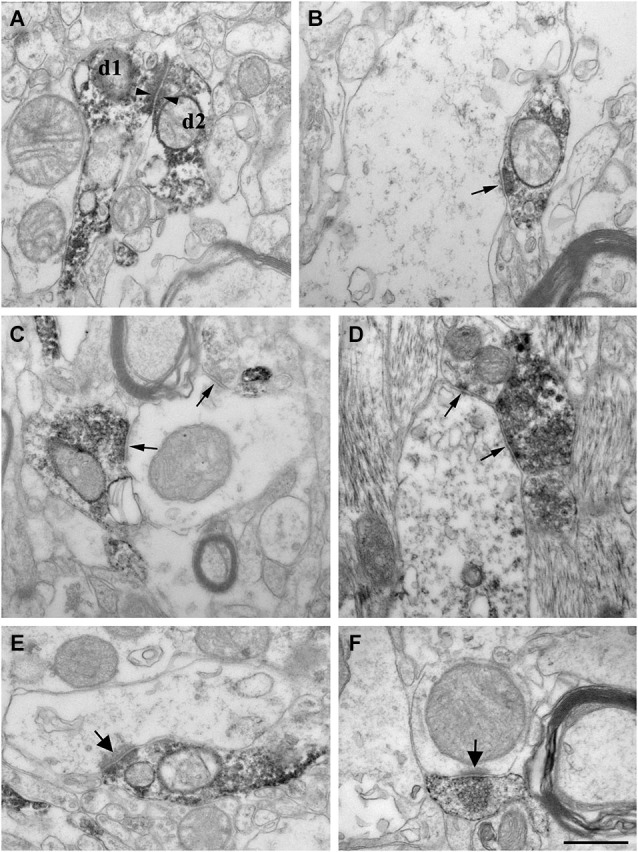
**High power electron micrographs from control (A, B, E) and epileptic CA1 region (C, F-non sclerotic, D-sclerotic)**. In the control samples, dendrites of CR-positive interneurons are often attached to each other by zona adherentia-type contacts (d1 and d2 in **A**, arrowheads). Terminals of CR-positive interneurons establish symmetric synaptic contacts (**B**, **C**, **D**, arrows). In the control CA1 region they frequently terminate on pyramidal cell dendrites **(B)**. In the non-sclerotic epileptic samples, unlabeled interneuron dendrites are more often targeted **(C)**. Almost all of the targets belonged to interneuron dendrites in the sclerotic CA1 region **(D)**. In addition, CR-positive terminals giving asymmetric synaptic contacts (presumably excitatory) were also frequently observed both in control (**E**, arrow) and epileptic samples (**F**, arrow). Most of them were located in the stratum lacunosum-moleculare. Scale: 500 nm.

In controls, the majority of the CR-positive inhibitory axon terminals contacted CR-positive interneurons and pyramidal cells in the CA1 region (Figure 5; Tóth et al., 2010). In the epileptic samples, the proportion of CR-positive targets was significantly reduced. The decreased innervation of other CR-positive dendrites may reflect the impairment of the CR-containing interneuronal network. The ratio of unstained interneuron dendrites increased among the targets, whereas pyramidal cells were less frequently innervated, even in those patients where the pyramidal cells were present (non-sclerotic patients) (Figure 5; Tóth et al., 2010). The frequency of CR-positive terminals giving symmetric synapses was decreased, whereas those giving asymmetric synapses was increased in the epileptic CA1 region (Tóth et al., 2010).

Taken together, these results suggest that both synaptic and dendro-dendritic contacts of CR-positive interneurons are impaired even in the non-sclerotic epileptic human hippocampi, in the absence of any major principal cell- and CR-containing interneuron loss (Tóth et al., 2010).

## Changes to the extrinsic CR-containing system in human epileptic hippocampus

Besides the changes affecting the intrinsic CR-containing system, the extrinsic CR-expressing pathways also show alterations. In the epileptic dentate gyrus, the CR-containing excitatory pathway—originating from the supramammillary nucleus—is expanded to the outer two thirds of the molecular layer (Figure 3; Maglóczky et al., 2000). This observation was confirmed at the electron microscopic level: whereas in controls the majority of the CR-positive excitatory terminals were located in the inner molecular layer, the frequency of terminals was similar in the inner and outer moleculare in the epileptic cases (Maglóczky et al., 2000). The extension of the supramammillary pathway was independent from the granule cell dispersion, as it occurred in all the subjects examined. The relative increase of the frequency of CR-positive terminals giving asymmetric synapses in the CA1 region could also reflect the sprouting of an excitatory pathway originating presumably from the thalamus (Amaral and Cowan, 1980; Bokor et al., 2002).

## Epilepsy models and the fate of CR-containing interneurons

Numerous models of epilepsy were developed to study the mechanisms of seizures and epilepsy including genetic models, *in vitro* slice models, *in vivo* induced seizures, acquired focal models etc. (Pitkanen et al., 2006). In most cases rodents were used for *in vivo* chronic models, and the cortical and hippocampal areas were especially studied.

The pathological changes of the brain were classified and the sensitivity of neurons was monitored in most models. It was shown that GABAergic cells are more preserved than principal cells (Babb et al., 1989; Davenport et al., 1990a; Houser, 1991; Ben-Ari, 2001; Ben-Ari and Holmes, 2005). However, certain neurochemically identified groups of interneurons proved to be sensitive to epileptic injury, like somatostatin cells in TLE (de Lanerolle et al., 1989; Mitchell et al., 1995; Sundstrom et al., 2001). Interestingly, as compared to other GABAergic interneurons, the fate of CR-containing interneurons was less examined in epilepsy.

**Figure 6:**
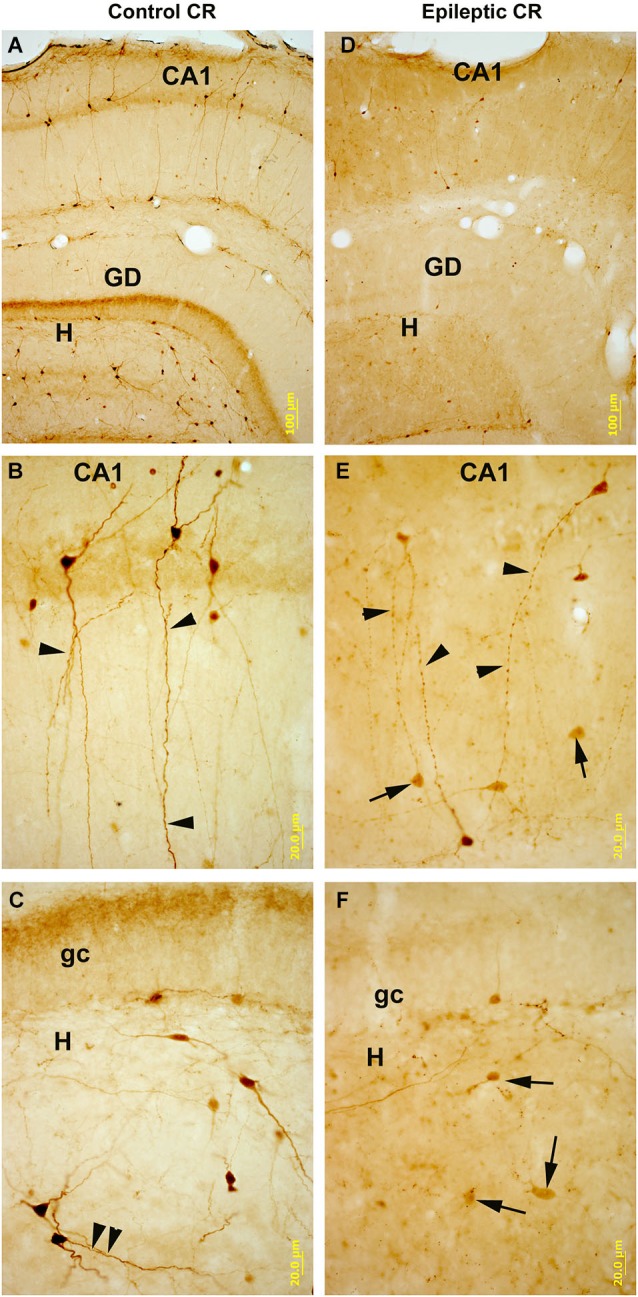
**CR-immunostained sections of hippocampal subfields in control and kainate-treated epileptic rats after one weak survival time**. In control **(A–C)** CR-containing interneurons are numerous in all subfields of the hippocampus. They are heterogeneous in shape and localization (panel **A**), they posses smooth or sparsely spiny dendrites. In the CA1 region bipolar cells are dominant with long, parallel running dendrites (panel **B**). Arrowheads point to the dendritic contacts. In the hilus (H) of the dentate gyrus (DG, panel **C**) they are mostly multipolar cells, and parallel running dendrites are also present here (arrowheads). In the hippocampi of the kainate-treated rats **(D–F)** the number of CR-containing cells is profoundly decreased in all subfields. In CA1 (panel **E**) the surviving cells have segmented, varicose dendrites (arrowheads). In the hilus the decrease in the number of cells is more severe than in the CA1. Arrows point to the dying, faint cells with reduced/absent dendritic tree. DG: dentate gyrus; gc: granule cell layer; H: hilus. Scales: **(A,D)**: 100 um; **(B–F)**: 20 um.

Hippocampal CR-positive cells were shown to be vulnerable to excitotoxic cell damage in ischaemia (Freund and Maglóczky, 1993) and in various models of epilepsy (Figure 6; Magloczky and Freund, 1993; Maglóczky and Freund, 1995; Ben-Ari and Cossart, 2000; Bouilleret et al., 2000; André et al., 2001; Domínguez et al., 2003; Slézia et al., 2004; van Vliet et al., 2004; Cobos et al., 2005; Tang et al., 2006; Wu et al., 2012; Huusko et al., 2013).

### Similarity to human TLE

For a detailed comparison to human TLE, data from chronic models of TLE concerning the CR-positive interneurons is summarized here. These models usually show remarkable similarity to human hippocampal pathology in TLE (Sloviter, 1996; Bernard et al., 1998; Ben-Ari, 2001; Sharma et al., 2007; Curia et al., 2008; Tang and Loke, 2010; Carriero et al., 2012), including sclerosis, loss of pyramidal cells in the CA1 and CA3c regions, hilar interneuronal loss, and the preservation of calbindin-containing interneurons (Sloviter et al., 1991; Wittner et al., 2002).

Reduction in the number of CR-containing cells was found in the hippocampus after electrical induction of status epilepticus (van Vliet et al., 2004), in traumatic brain injury (Huusko et al., 2013), in a kainate model of TLE (Figure 6; Magloczky and Freund, 1993; Maglóczky and Freund, 1995) and in the pilocarpine model of epilepsy (André et al., 2001; Tang et al., 2006; Zhang et al., 2009; Wu et al., 2012). CR-positive cells showed sensitivity for single status epilepticus without recurrent seizure (Fabene et al., 2001).

Although CR-immonopositive interneurons are morphologically and functionally different (Gulyás et al., 1992), no selective loss of a certain morphological type was found in the models of epilepsy. The spiny CR-positive cells in the stratum lucidum of CA3 regions showed profound sensitivity, as they die both in ischaemia (Freund and Maglóczky, 1993) and epilepsy (Magloczky and Freund, 1993; Maglóczky and Freund, 1995; André et al., 2001; Domínguez et al., 2003; Zhang et al., 2009) presumably due to their strong mossy fiber input, which is further enhanced in epilepsy. The bipolar cells—presumably responsible for interneuron specific inhibition—are also strongly decreased in number, and their dendritic tree is reduced in size and shows segmentation in epileptic hippocampi (Figure 6; Magloczky and Freund, 1993; Slézia et al., 2004; Zhang et al., 2009), similarly to that found in human TLE (Tóth et al., 2010). Besides synaptic contacts, CR-immunopositive cells are also connected by their dendritic tree (Figure 6; Gulyás et al., 1992), and therefore the disruption of the dendritic connections may cause dysfunction of these cells in rodent models.

Interestingly, even a small dose of kainate (Maglóczky and Freund, 1995) may cause reduction in the number of CR-containing cells, especially in the hilus, suggesting that hilar CR-positive interneurons may be more sensitive than neurons in other subfields (Figure 6).

Analyses of the time course of the cell loss showed that it begins soon after excitotoxic insults (Freund and Maglóczky, 1993; Maglóczky and Freund, 1995). After few hours, a significant decrease can be seen in the hilus and CA3 of the rat.

However, granule cells and mossy cells also contain CR in mice (Liu et al., 1996; Fujise et al., 1998; Mátyás et al., 2004), especially in young animals (Blasco-Ibáñez and Freund, 1997; Brandt et al., 2003). In older animals the CR-staining of the principal cells is weaker. In addition, induction of status epilepticus may transiently accelerate the production of newly formed neurons (Kralic et al., 2005), which are mostly granule cells (Hester and Danzer, 2013). Therefore, more numerous CR-positive cells might be seen in the epileptic mice after pilocarpine-induced epilepsy, their number is decreased at the chronic phase with the decreasing tendency of neuron production (Kralic et al., 2005) and weakening of the CR-immunopositivity of principal cells with time (Brandt et al., 2003).

### Mechanism of death of CR-containing neurons

The exact mechanism of death of CR-containing cells is not known. According to the morphological signs (Martin, 2001), CR-containing cells die by a necrotic type of excitotoxic degeneration, not by apoptosis. Their electron microscopic examination shows degenerating cytoplasm, decaying mitochondria and numerous phagocytic vacuoles, suggesting the overproduction of abnormal proteins and energy failure (Maglóczky et al., 2000; Tóth et al., 2010).

Although these cells contain a calcium binding protein, they proved to be extremely sensitive for epileptic and ischemic conditions with large calcium ion influx. One explanation of their extreme sensitivity can be their strong connectivity with each other, as they may react to the insults as a network (Gulyás et al., 1992; Tóth et al., 2010). On the other hand, they contain thin cytoplasm and they are poor in organelles. Therefore we can assume that their energy supply is weaker and makes them vulnerable to excitotoxic stress (Hipólito-Reis et al., 2013). The increased excitability of CR-positive cells in epilepsy, caused by the upregulation of a voltage-gated Na channel (Kim et al., 2008) may also contribute to their vulnerability to excitotoxic insults.

## Conclusion—consequences of the vulnerability of CR-positive interneurons

The alterations and/or sensitivity of different calcium-binding protein-containing interneurons in human TLE were discussed in several reports (Sloviter et al., 1991; Maglóczky et al., 2000; Wittner et al., 2001, 2002; van Vliet et al., 2004; Tóth et al., 2010). Calbindin-containing interneurons seem to be preserved and enlarged, and display a sprouted axonal arbor. The parvalbumin-containing cells are also preserved, although many of them most likely loose immunoreactivity for parvalbumin due to calcium-overload (Johansen et al., 1990). In contrast, CR-containing interneurons are highly sensitive. The different vulnerability of these calcium-binding protein containing interneurons probably depends on the distinct input-output properties of these cells and the intrinsic enzymatic properties, number of mithochondria etc., rather than the type of the calcium-binding protein they express. CR-containing interneurons were shown to be vulnerable in epilepsy in most published studies. Decreased cell number (Magloczky and Freund, 1993; Maglóczky and Freund, 1995; André et al., 2001; Slézia et al., 2004; van Vliet et al., 2004; Tang et al., 2006; Muzzi et al., 2009), altered dendritic tree, decreased amount of dendritic contacts between cells (Tóth et al., 2010) and a synaptic reorganization of CR-positive inhibitory terminals were described (Maglóczky et al., 2000; Tóth et al., 2010).

These results are of special interest, since dendritic inhibition was shown to be impaired in epilepsy, together with an intact perisomatic inhibition (Cossart et al., 2001). This can be only partially explained by the sensitivity of somatostatin and neuropeptide Y-containing dendritic inhibitory interneurons (de Lanerolle et al., 1988; Mitchell et al., 1995; Sundstrom et al., 2001), since the axons of these cells show a remarkable sprouting (de Lanerolle et al., 1989; Sperk et al., 1992). In addition, the well preserved (Sloviter et al., 1991) calbindin-containing dendritic inhibitory cells also show an axonal sprouting (Wittner et al., 2002). However, despite of the sprouting of dendritic inhibitory cells, dendritic inhibition of pyramidal cells was found to be ineffective in TLE (Cossart et al., 2001; Ben-Ari and Dudek, 2010).

Taken together, these findings suggest that the sensitivity of CR containing interneurons plays an important role in the impairment of dendritic inhibition in epilepsy:

1. the synchronous activation of these interneuron-selective inhibitory cells is possibly impaired, leading to an asynchronous and less effective dendritic inhibition
2. the CR-containing population of dendritic inhibitory cells are also impaired, further decreasing the efficacy of dendritic inhibition
3. due to the changes in their synaptic target distribution, they innervate principal cell dendrites less frequently in epileptic human hippocampus

Since the impaired dendritic inhibition may cause a less effective control of the efficacy and plasticity of excitatory inputs to principal cells, and subsequently to the formation of principal cell assemblies connected by abnormally potentiated synapses, the impairment of CR-positive cells can be involved in epileptogenesis and seizure generation (Maglóczky and Freund, 2005; El-Hassar et al., 2007; Tóth et al., 2010). Furthermore, these excitatory inputs were shown to undergo sprouting both in animal models (Perez et al., 1996; Esclapez et al., 1999; Ben-Ari, 2001; Lehmann et al., 2001) and humans (Lehmann et al., 2000; Magloczky, 2010), partly explaining why severe intractable seizures can occur even in non-sclerotic patients, where the majority of principal and non-principal cells are preserved (Maglóczky and Freund, 2005; Magloczky, 2010; Tóth et al., 2010). The excessive reorganization of the hippocampal inhibitory network, the sprouting of either intrinsic or afferent excitatory pathways, together with the intact output from the CA1 region, may render the non-sclerotic hippocampus a potent epileptogenic region (Maglóczky and Freund, 2005; Magloczky, 2010).

The sensitivity of CR-expressing interneurons for excitotoxic insults was also shown in animal models (Magloczky and Freund, 1993; Maglóczky and Freund, 1995; André et al., 2001; Domínguez et al., 2003; Zhang et al., 2009). Since the early loss of these cells can be observed in the acute and latent phase of epileptogenesis (Zhao et al., 2008), we can hypothesize that recurrent seizure generation might be associated with a loss of a certain amount of CR-positive cells, among other factors.

Prevention of the loss of CR-positive cells and preserving the integrity of the attached CR-positive dendrites may have antiepileptic effects, protecting the proper inhibitory function and helping to spare principal cells in epilepsy (Tóth et al., 2010). Since they die by degeneration after excitotoxic insults in the acute-to-latent phases, one therapeutic possibility may be to promote their survival during the initial phase of epilepsy.

## Conflict of interest statement

The authors declare that the research was conducted in the absence of any commercial or financial relationships that could be construed as a potential conflict of interest.

## Abbreviations

CA 1, 2, 3: regions of the Cornu Ammonis according to Lorente de No
CR: calretinin
TLE: temporal lobe epilepsy.
